# Stroke and Falls—Clash of the Two Titans in Geriatrics

**DOI:** 10.3390/geriatrics1040031

**Published:** 2016-11-30

**Authors:** Kit Mun Tan, Maw Pin Tan

**Affiliations:** 1 Division of Geriatric Medicine, Department of Medicine, Faculty of Medicine, University of Malaya, Kuala Lumpur 50603, Malaysia; kmtan@ummc.edu.my; 2 Ageing and Age-Associated Disorders Research Group, Faculty of Medicine, University of Malaya, Kuala Lumpur 50603, Malaysia

**Keywords:** stroke, accidental falls, aged

## Abstract

Both stroke and falls are common conditions affecting the older adult. Despite stroke being considered a well-established major risk factor for falls, there remains no evidence for effective prevention strategies for falls specifically for stroke survivors. Previous observational studies evaluating falls risk factors in stroke have mainly been uncontrolled and found similar risk factors for stroke populations compared to general older populations. Specific risk factors, however, are likely to play a greater role in stroke survivors including unilateral weakness, hemisensory or visual neglect, impaired coordination, visual field defects, perceptual difficulties and cognitive issues. In addition, individuals with stroke are also more likely to have other associated risk factors for falls including diabetes, falls risk increasing drugs, atrial fibrillation, and other cardiovascular risk factors. While anticoagulation is associated with increased risk of intracranial bleeding after a fall, the risk of suffering a further stroke due to atrial fibrillation outweigh the risk of bleeding from a recurrent fall. Similarly, while blood pressure lowering medications may be linked to orthostatic hypotension which in turn increases the risk of falls, the benefit of good blood pressure control in terms of secondary stroke prevention outweighs the risk of falls. Until better evidence is available, the suggested management approach should then be based on local resources, and published evidence for fall prevention. Multicomponent exercise and individually tailored multifactorial interventions should still be considered as published evidence evaluating the above have included stroke patients in their study population.

## 1. Introduction

The risk of stroke and falls both increase with age. In addition, stroke is an established risk factor for falls with up to 37% of all stroke survivors reporting at least one fall in the preceding six months [1]. A recent study, however, found no increased risk of falls when comparing stroke participants with non-stroke controls. This study also revealed that stroke participants were more likely to suffer both recurrent falls and fear of falling [2]. Despite both these condition being highly prevalent among older adults, the relationship between falls and stroke remains understudied. In addition, few studies to date have addressed specific risk factors for falls in stroke and effective management strategies for falls prevention in stroke.

This clinical review presents published evidence for the epidemiology, risk factors, prevention and management of post-stroke falls. We will also propose a rational approach to the evaluation for falls risk and complications, as well as suggested topics for future research. Contentious issues such as anticoagulation and antihypertensive treatment will also be addressed.

## 2. Methods

We searched PubMed and Google Scholar for English Language articles published after 2006, with titles, abstracts and keywords containing the words: (“stroke” OR “cerebrovascular disease”) AND (“accidental fall*” OR “fall*”). Additional articles were also obtained through the authors’ personal archives and cross-referencing.

## 3. Epidemiology of Falls in Stroke

The incidence of falls after stroke reported in the literature varies with patient selection, geographical location and duration after stroke. A recently published community-based study involving 567 individuals with acute stroke in North Dublin established an incidence of 1.50 per 1000 person-years, with 23.5% of their stroke population reporting at least one fall in two years [3]. In a study conducted among consecutive admissions to an inpatient rehabilitation unit, 37% of inpatients had fallen at least once in the duration of their stay [4]. Recent studies evaluating falls occurrence over shorter periods of six months have found that stroke survivors may not necessarily fall more frequently than non-stroke older adult populations as previously suggested [1,2,5,6]. This may be because those with more severe strokes may paradoxically fall less frequently due to severe limitations to their mobility, which may then potentially mask the increased risk of falls associated with less severe strokes [7]. Individuals with severe disability due to stroke are also likely to have increased mortality. Longer term studies, however, clearly demonstrate increased risk of falls among stroke survivors, with stroke survivors twice as likely to experience falls than non-stroke controls after a median of 10 years post-stroke [8,9]. Regional variations, however, may also explain lack of increase in falls incidence with stroke in recently published studies, with the newer studies being conducted predominantly among Asian populations [2,5,6], and historical studies reporting increased prevalence published from predominantly Caucasian populations [1]. This raises a possibility of cultural differences in falls risk, though Callaly’s study also inferred no increased incidence of falls with stroke [3]. 

While recent studies now raise doubts about the previously reported increased incidence of falls after stroke over shorter terms of two years or less, published studies do suggest that stroke is associated with increased falls frequency or increased risk of recurrent falls [2,9]. Jorgensen et al. who compared falls in long term stroke survivors and non-stroke controls found a three-fold increase in risk of recurrent falls [8].

## 4. Risk Factors

Studies evaluating risk factors for falls in stroke have mainly been conducted exclusively among stroke survivors with few studies embarking on comparisons between risk factors among stroke and non-stroke populations to identify potential differences. The risk factors identified for falls in stroke are similar to those of falls in the general population [10,11]. Falls in stroke survivors are therefore usually attributed to a combination of factors which may or may not be related to stroke, and stroke is only one of the major comorbidities affecting the older adult. The rest of this section will highlight the scant evidence on stroke-related risk factors for fall. In addition, potential risk factors that could be considered of higher importance in stroke will be extrapolated from established knowledge of risk factors for falls among older persons in general.

### 4.1. Stroke-Related Deficits

#### 4.1.1. Motor Deficits

It is common practice to assume that motor weakness would be the primary reason why the stroke survivors should fall. While muscular weakness is a major risk factor for falls, the deficits associated with stroke are highly variable between each patient and stroke event, and highly dependent on the underlying stroke etiology and severity of stroke [7]. While motor deficits can occur in isolation in those with lacunar strokes, individuals with anterior circulation strokes will have other associated deficits, while in those with posterior circulation strokes, it is the coordination rather than weakness that is affected which then leads to major risk of falls due to balance issues. Therefore, in estimating fall risk, measures such as functional mobility, which had been found to be associated with fear of falling in stroke survivors, is potentially more useful than identifying individual deficits [2]. However, previous studies indicated that persons with poorer upper limb function may be more likely to fall due to the inability to save themselves when falling [12,13].

#### 4.1.2. Mobility and Dependency Level Post-Stroke

Callaly et al. found that persons with a Modified Rankin Scale (MRS) of 2–3 at 90 days were at highest risk of falling two years post-stroke, whereas those with a MRS of 4–5 were at intermediate risk compared to stroke patients who could mobilize independently [3].

#### 4.1.3. Sensory

Sensory deficits are often overlooked in stroke, and the role of this in falls post-stroke remains unclear. It is expected that as sensation plays a vital role in maintaining postural stability, sensory deficits will indeed lead to an increase in risk of falls post-stroke. Hemi-sensory neglect is also a common feature of anterior circulation strokes, particularly those with non-dominant hemisphere strokes. The associated anosognosia or lack of awareness of their physical weakness as well as potential dangers in their physician surroundings is a particular danger [14,15]. Individuals with right parietal lobe infarcts, for instance, may present late with falls rather than hemiparesis, as the individual is unaware of the apparent physical deficit due to the associated hemi-inattention.

#### 4.1.4. Visual

Visual field defects, visual hemi-inattention, diplopia and cortical blindness may all occur as a result of stroke, all of which will require different management strategies [16].

#### 4.1.5. Perception/Abstract Thinking

Cortical strokes affecting relatively silent areas of brain may also lead to delayed presentation, with falls or acute confusional state. Loss of sequencing or problem solving abilities will lead to difficulties in negotiating physical obstacles or performing daily tasks in a safe and effective manner [17,18]. Little is currently known about these higher cortical functions and their relationship with falls post-stroke.

#### 4.1.6. Cognitive Function

Cognitive function is often affected, and tend not to be regularly evaluated after stroke, with the main focus of management being on restoring function. However, even mild cognitive loss is likely to lead to impaired judgment, gait disorders and reduced ability to dual-task [19]. A study by Alemdaroglu et al. found that patients with left hemispheric stroke were more like to fall within six months of discharge from a rehabilitation hospital than patients with right hemispheric stroke [20]. They offered the explanation that persons with right-hemispheric lesions tend to be better supervised given their impaired judgment and lack of insight into their problems, compared to persons with left hemispheric lesions who usually have communication deficits but intact visuomotor perception and memory, with some ability to learn by demonstration and to synthesize part of the task. This can cause them to appear to be more independent functionally leading to a lower level of supervision. However there were previous studies that showed that patients with right hemispheric strokes were at higher risk of falls than left in patients who were able to walk independently and in repeat fallers [21], related to perhaps the sensory neglect and other cognitive deficits of a right hemispheric stroke.

#### 4.1.7. Cerebellar and Vestibular Function

Posterior circulation strokes may lead to either cerebellar or vestibular deficits. The associated symptoms of vertigo are often distressing and difficult to control pharmacologically. Position sense and coordination, both essential components of balance, if impaired leads to compromised ability to maintain the upright posture, despite preservation of motor control [22].

### 4.2. Cardiovascular Risk factors

#### 4.2.1. Atrial Fibrillation

An increasing proportion of strokes are now being attributed to atrial fibrillation (AF) [23]. The presence of AF, however, is also associated with bradyarrhythmias from sick-sinus syndrome or rate-reducing drugs [24]. In addition, with the strong evidence and increased awareness for the benefits of anticoagulation for stroke prevention, individuals with AF are also often anticoagulated [25]. This will be discussed in the next section. The risk of carotid sinus hypersensitivity (CSH) also increases with the presence of AF. Bradycardia and or hypotension in response to carotid sinus stimulation is observed in CSH, with the cardioinhibitory subtype observed in up to 23% of unexplained falls presenting to the emergency room [26].

#### 4.2.2. Hypotensive Disorders

As both stroke and syncope are age-related disorders, falls related to syncope can co-exist with stroke-related falls. Little is currently known about the potential interactions between stroke and syncope. In addition to CSH, hypotensive disorders such as vasovagal syncope (VVS) and orthostatic hypotension (OH) may be more common following stroke due to primary autonomic nervous system involvement [27], the adverse effects of medication or the presence of other related diseases such as diabetes. Furthermore, the presence of stroke disease will increase the likelihood of development of symptoms with CSH and OH, which often occurs asymptomatically among older adults with manifestation of symptoms once cerebral autoregulation is impaired [28]. In addition, the individual may not recall prodromal or syncopal symptoms from OH, VVS or CSH due to lack of awareness or amnesia for loss of consciousness [29,30,31].

### 4.3. Psychological Risk Factors

Depression is a common complication of stroke as well as a well-established risk factor for falls in the general older population [8]. The mechanisms by which depression leads to falls have yet to be adequately characterized. Furthermore, the use of antidepressants is also associated with increased risk of falls.

Fear of falling, commonly assigned the term post-fall syndrome, had been considered a common and devastating consequence of falls leading to activity avoidance, social isolation and physical and psychological deterioration. However, in stroke, fear-of-falling is found to be highly prevalent regardless of the prevalence of falls. It is not entirely clear whether fear-of-falling is linked to increased risk of falls in stroke [32,33].

### 4.4. Medications

#### 4.4.1. Blood Pressure Lowering Therapy

Blood pressure (BP) lowering therapy is now routinely prescribed post-stroke to those with at least Grade 1 hypertension (systolic BP > 140 mmHg and diastolic BP > 90 mmHg) [34]. Conversely, doctors have tended to discontinue BP lowering therapy among older adults with falls due to concerns with regards to OH [35]. However, emerging evidence now suggests that the use of BP lowering therapy alone may not be associated with increased risk of falls [36]. Though, increased cumulative antihypertensive burden calculated based on the daily defined dose, is linked to fall recurrence in the general older population [37]. Callaly et al. did however find that post-stroke patients treated with an alpha-blocker were at increased risk of falls within two years of their stroke [3]. The stroke risk associated with withholding or discontinuation of BP lowering therapy after a fall needs to be evaluated carefully in terms of risk to benefit ratio, but alas, there has been no published evidence so far to direct decision making in this area. Available guidelines have suggested individualized BP treatment targets to be prescribed by experienced physicians [34].

#### 4.4.2. Anticholinergic Burden

Recent evidence has linked anticholinergic properties of both prescribed and over-the-counter pharmacotherapy with increased risk of both stroke and falls [38,39,40,41]. Acetylcholine is a peripheral and central neurotransmitter which facilitates smooth muscle action, secretory function as well as central processing. The inhibition of cholinergic receptors leads to the adverse effects of mental confusion, blurred vision, dry mouth, muscle weakness, urinary retention and constipation. Over-the-counter medications such as cold remedies and antihistamines have been found to contain anticholinergic action. Drugs often used to alleviate stroke complications, including oxybutynin and baclofen for bladder instability and spasticity, respectively, have strong anticholinergic effects.

#### 4.4.3. Falls Risk Increasing Drugs

The list of falls risk increasing drugs include medications commonly prescribed in the management of stroke and stroke complications, namely antidepressants and antiepileptics [42]. The caveats would be that both depression and epilepsy are common complications post-stroke that if untreated also lead to falls [43,44].

#### 4.4.4. Anticoagulation

While the use of anticoagulants per se do not influence falls risk, anticoagulants are commonly discontinued after falls to avoid potential bleeding complications after falls including subdural hematoma, intra-abdominal bleeding and uncontrollable bleeding from fracture and soft tissue injury [45]. However, particularly in patients with AF, the risk of stroke recurrence is high, and usually likely to outweigh the risk of bleeding, though risk to benefit ratios need to be considered on an individual basis, and in individuals with other bleeding diathesis or recurrent subdural hemorrhage for instance, the risk of bleeding may indeed be too high [46]. A review by Garwood et al. found that the benefits of anticoagulation still outweighed the risks in patients with atrial fibrillation who fall [47]. Patients need to be carefully counseled, and all possible measures taken to minimize falls risk in those anticoagulated for AF post-stroke. At the same time, all modifiable risk factors that can increase the risk of bleeding in anticoagulation in AF should be addressed or reduced in all patients, especially in patients who have an increased risk of fall. The HASBLED score is the validated score most in use at present to determine risk of bleeding on anticoagulants [48]. Although the HASBLED score does not include risk of falls, the score can be used to identify potentially modifiable risk factors for bleeding while on anticoagulation for AF.

### 4.5. Epilepsy

Individuals with post-stroke epilepsy occasionally present with falls, which are often unwitnessed [49]. The possibility of epilepsy therefore should be considered in those presenting with falls post-stroke, and careful history taking including a witness history obtained as far as possible. Features suggesting the presence of epilepsy include telltale prodromal symptoms and prolonged confusion after falls.

## 5. Investigative Strategy

### 5.1. Post-Stroke Falls Prediction

A recent systematic review has identified several published prediction models for falls which mostly included hemi-inattention for in-patient falls, and history of falls and balance measures for community studies [50]. These instruments currently have limited clinical applicability, and further research is urgently required. The presence of multiple risk factors associated with falls should raise concerns for falls risk and be addressed with appropriate prevention strategies.

### 5.2. Investigation of Falls Post-Stroke

**Figure 1:**
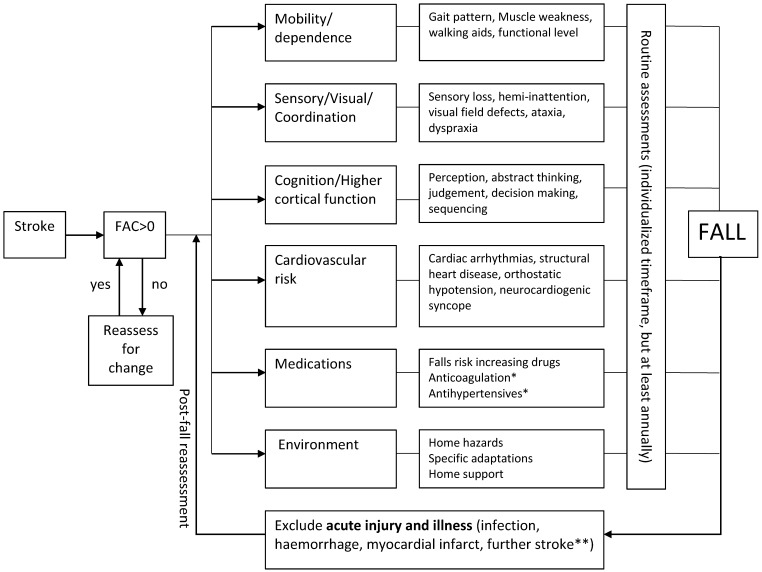
Evaluation of Falls Risk Post-Stroke. Flowchart demonstrated a suggested universal approach to fall risk assessment among all individuals with stroke. *, treatment benefit usually exceeds risk; **, new focal neurological deficits should exist; FAC, functional ambulation category.

There is currently inadequate published evidence to support specific recommendations for the investigations of falls for stroke survivors. However, as previous literature have highlighted similar risk factors in stroke and general adult populations, the investigative strategy barring a few exceptions should not differ from that of general older adult populations (Figure 1). A detailed history to establish the circumstances of the fall in as much detail as possible should be obtained. Whenever possible, a witness history should also be obtained. Existing guidelines suggest that gait and balance assessments are conducted after the first fall, and the remaining assessments should only be conducted if falls are recurrent [51]. As stroke survivors are at greater risk of recurrent falls, the initiation of other investigations should perhaps occur after the first fall, rather than waiting for a further fall to occur, even when there is no gait and balance abnormality. Other assessments to be considered include for home hazards, medication review, visual assessment, cognitive testing, and psychological assessment.

### 5.3. Baseline Investigations

All stroke survivors who present immediately after a fall should first of all be evaluated for the presence of injury and other complications such as pneumonia. In addition, falls may also be precipitated by acute intercurrent illness such as acute gastrointestinal bleeding, urinary tract infection, myocardial infarction and decompensated heart failure, which could be life-threatening. All fallers should be investigated with a 12-lead electrocardiogram and postural blood pressure measurements. Postural BP measurements should be measured with non-invasive continuous beat-to-beat digital blood pressure measurements if available. Supine measurements should be recorded after at least 5 min of supine rest [52]. If automated oscillometric or manual sphygmomanometric measurements are used, the standing BP should be measured at two and three minutes after assuming the upright posture [53]. When using continuous digital continuous BP measurements, traditional cut-off values of 20 mmHg systolic and 10 mmHg diastolic BP reduction with standing for OH may be too low. Adopting a higher cut-off of 30 mmHg systolic BP drop may be more specific, but lacks sensitivity [31]. Measurement of postural BP with oscillometric or manual BP measurements may conversely miss large transient drops in blood pressure. Furthermore, a recent study has found that standing BP measured at two or three minutes after standing may be a more useful measure, though an appropriate lower cut-off BP value beyond which falls are more likely remains elusive [53].

### 5.4. Gait and Balance

The assessment of gait and balance is of vital importance. Visual observation of gait will reveal gait abnormalities associated with stroke such as the high-stepping gait or the broad-based gait. To improve objectivity, and to aid future monitoring of progress, validated physical assessment tools should be used. The Functional Ambulation Category is a commonly used measurement of walking ability post-stroke [54]. Measurements falls investigators are more familiar with are the Timed-up-and-Go or Get-Up-and-Go test, Tinetti’s Gait and Balance Scale, and the Berg’s Balance Scale [55,56,57]. The above scales have been evaluated as falls risk screening instruments. Most are measures of functional mobility, of which other validated measures do exist, all of which measure similar constructs with no absolute advantage of one above the other [58].

### 5.5. Home Hazards

Home hazards assessments should be performed by trained occupational therapists (OT) if possible, but non-OT tools are a trade-off in settings where OT services are not available [59]. Environmental assessments are often conducted as part of the discharge process after a stroke, particularly for those who undergo a period of inpatient rehabilitation. This should be prioritized for those deemed at higher risk of falls [60].

### 5.6. Cardiovascular Assessment

In individuals with unexplained falls the presence of cardiac or neurocardiogenic syncope should be considered. An abnormal ECG and history of heart disease should raise the suspicion of cardiac syncope. Ambulatory ECG measurements should then be obtained [61]. While 24 h Holter measurements are the most widely available, the diagnostic yield is low, with the test only likely to be positive should falls occur practically daily. Longer term external loop recorders may have a role in the evaluation of falls which occur at least one a month. Early insertion of an implantable loop recorder may be cost-effective with improved diagnostic yield [62]. Echocardiography should be considered if there is clinical evidence of heart failure or an audible murmur. In addition, the 12-lead ECG and the history may suggest ischemia or exercise related arrhythmia in which case cardiac stress tests, such as an exercise test if possible, stress echocardiogram or nuclear stress test should be considered, and an angiogram conducted if the former suggested the possibility of reversible ischemia [63].

In individuals with unexplained falls, carotid sinus massage or tilt-table testing should be considered to determine the presence of CSH or vasovagal syncope. Previous reports have warned against a small risk of stroke with carotid sinus massage used to diagnose CSH, but a recent multi-center study involving 1401 patients has reported no stroke complications with carotid sinus massage [64]. These investigations usually emphasize the reproduction of symptoms of syncope or presyncope in normal adult populations [65]. However, it would be wise to exercise greater caution in the stroke population and to avoid prolonged hypotension, at the expense of achieving a confirmatory diagnosis. The diagnosis may therefore have to be made based on the finding of hypotensive responses to carotid sinus massage or tilt-table testing, in the absence of plausible diagnosis after further investigation.

### 5.7. Imaging

The role of brain imaging in post-stroke falls is often a source of potentially avoidable healthcare expenditure, with recurrent fallers sometimes subject to several CT scans within a year. The main reason for conducting brain CT scans after a fall is to detect subdural hematomas. Therefore, brain scans should be conducted should there be a history of head injury with loss of consciousness, reduced consciousness, signs of raised intracranial pressure, seizures or new focal neurological deficits on presentation [66]. Many clinicians would also consider an urgent brain CT essential if the patient has been on long term anticoagulation therapy. It is unusual, however, for falls to occur as the result of an acute stroke without obvious new neurological deficits. Therefore, CT brain scans in individuals without altered consciousness or focal neurological deficits are usually unwarranted.

### 5.8. Other Investigations

There is no established role for carotid Doppler ultrasound in the investigation of post-stroke falls. Transient ischemic attacks present with transient unilateral symptoms and signs with complete recovery, rather than falls. However, in individuals who are investigated with Doppler ultrasound as part of their post-stroke work-up, the presence of bilateral tight stenosis may discourage the physician from tight blood pressure control, though there is currently no evidence to support the concerns with regards to cerebral perfusion and subsequent falls. The role of EEG is only limited to those we suspect epilepsy. However, the inter-ictal EEGs may offer no additional diagnostic information above a good history from both the patient and a witness. Nevertheless, the sensitivity and specificity of EEG are unchanged among older adults compared to younger individuals [67].

## 6. Management

**Figure 2:**
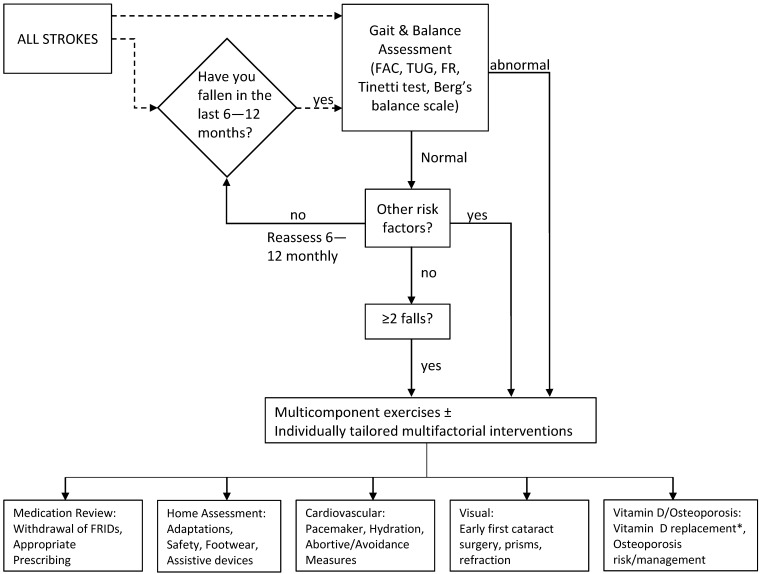
Management of Post-stroke Falls. Flowchart outlining suggested management strategy for primary and secondary prevention of post-stroke falls. Fenestrated line suggests adaptation according to locally available resources. * Only if proven deficiency or limited sunlight exposure; FAC, functional ambulation category; TUG, timed-up and go; FR, functional reach.

There is to date, no published evidence for effective prevention strategies for falls in stroke [68]. The recommendations in this section are therefore based on extrapolated evidence from studies involving the general older population which shares similar risk factors with stroke fallers, with specific considerations in special situations associated with stroke (Figure 2).

Several potential approaches could be adopted in addressing falls prevention strategies. In the management of falls in the general population, clear distinctions are often drawn between primary and secondary prevention. If we were to base our recommendations on the body of evidence suggesting that stroke and cerebrovascular disease are established risk factors for falls, then the boundaries between primary and secondary prevention should be blurred in the stroke population, instead adopting blanket strategies for falls prevention measures regardless of falls history. However, with the recent publications suggesting that falls occurrence in stroke may not necessarily be as high as previously reported, perhaps an alternative approach of adopting published falls risk predictors to identify those most at risk and to concentrate resources on falls prevention in these individuals. In the absence of any clear evidence, we would recommend that the approach individual centers choose to adopt, i.e. universal vs. targeted approach, should be based on local availability of resources.

### 6.1. Physical Therapy

Stroke survivors should receive individualized physical therapy as part of their stroke rehabilitative process. The handful of published studies involving intervention studies, some of which were non-randomized, had evaluated sit-to-stand training, early mobilization, standing and sit-to-stand training, exercise training, and balance training, mostly showed no actual reduction in falls recurrence or fall frequency, with the two studies reporting statistical significance containing small samples sizes and hence requiring cautious interpretation [69]. There is therefore no evidence to suggest any additional physical interventions as primary prevention beyond the usual physical therapy already provided as part of stroke rehabilitation. However, in those with recurrent falls post-stroke, it may be worthwhile pursuing exercise therapy if the individual is physically able as a single intervention or as part of a multifactorial intervention. Previous studies reporting the effectiveness of exercise interventions had included stroke survivors, with Tai Chi and multicomponent physical exercise being most effective [70].

### 6.2. Medication Management

The benefits of medication review in falls reduction remains unclear even for the general older population. While numerous studies have linked falls risk increasing medications, potentially inappropriate prescribing and anticholinergic burden with falls, there is limited evidence that withdrawal subsequently reduces falls [71]. In addition, the withdrawal of these medications is often non-sustainable, with these medications often reinstated within a few months after withdrawal [72]. It is, however, reasonable to exercise caution with the prescription of falls risk increasing drugs, and if the use of these drugs are unavoidable, to prescribe them at the lowest dose and for the shortest possible treatment period. The withdrawal of blood pressure lowering therapy should only be carried out if there is clear evidence of over treated hypertension or a hypotensive disorder. This should be followed by careful monitoring, and, as far as possible, blood pressure control should still be aimed for, though actual blood pressure targets should be individualized [53].

### 6.3. Environmental Modification and Assistive Devices

Environmental or home modification should very much be part of the long-term rehabilitation process following a stroke and falls prevention measures should always be incorporated into this discharge process in those who are ambulatory. In addition to removal of loose mats and walkway clutter, installation of grab rails, appropriate lighting, etc., the use of assistive technology for fall detection and prevention should also be considered. These could take the form of bed or chair monitors, wearable devices, infrared monitors, alarm button, and the list goes on [73]. The latter is a rapidly advancing field and shows promise as accessible and affordable falls prevention measures of the future.

### 6.4. Cardiovascular Interventions

Hypotensive disorders including CSH, VVS and OH are often drug related, and can be treated by optimization of medications as well as conservative measures including avoiding potential precipitants, ensuring adequate hydration and taking immediate abortive actions. Occasionally, the implantation of permanent cardiac pacemakers may be necessary in those with evidence of bradyarrhythmia on the ECG recording, CSM or tilt-table [74]. Less frequently other cardiac or surgical interventions in the forms of angioplasty, cardiac stenting, bypass surgery and valve replacements may be curative.

### 6.5. Visual Interventions

Apart from early first cataract surgery, other studies on visual interventions in the general older population for the prevention of falls have yielded disappointing results [75]. A previous study had evaluated the use of Fresnel prisms in just 41 patients with visual neglect or hemianopia [76]. The results were equivocal, but little else can be drawn from the study due to its small sample size and few actual numbers who experienced subsequent falls.

### 6.6. Vitamin D and Osteoporosis Management

In the two systematic reviews on falls interventions in stroke published so far including 13 and 10 studies, only studies involving Vitamin D supplementation and osteoporosis treatment improved falls outcomes in stroke survivors [68,69]. Daily administration of 1000 IU of oral ergocalciferol in a randomized, placebo controlled study of hospitalized hemiplegic patients showing a large treatment benefit of 71% falls reduction [77]. The same group then conducted a further study comparing alendronate, a treatment for osteoporosis and the activated form of vitamin D, alphacalcidol, in institutionalized stroke survivors with immobilization induced hypercalcaemia, and demonstrated significant reduction in falls in the alendronate group [78]. The treatment benefit was surprising, as there is no clear mechanisms by which alendronate would reduce falls, as alphacalcidol may have conversely increased the risk of falls by worsening immobility associated hypercalcaemia. The above studies do not provide adequate evidence for routine use of vitamin D or alendronate for fall prevention in stroke. Two recent systematic reviews published in 2016 suggested an increased risk of hip fractures with stroke [79,80]. Individuals with stroke should be screened for osteoporosis risk factors and investigated with bone mineral density scans if found to be at increased risk and treated accordingly with bisphosphonates, teriparatide or denosumab. With emergent evidence linking strontium ranelate with increase in cardiovascular risk, strontium ranelate should be avoided among individuals with stroke [81]. Routine calcium supplementation should also be avoided with the recent evidence revealing the link with stroke-related dementia [82].

### 6.7. Multifaceted Interventions

Individualized multifactorial inventions have not been found to benefit individuals with stroke. Batchelor et al. evaluated the benefits of the modified Otago exercises in combination with falls education, falls risk minimization strategies and fracture minimization strategies in a randomized controlled study, and demonstrated no significant benefit with their multifactorial interventions [83]. However, multifactorial interventions have been widely promoted among older adults with recurrent falls, with published evidence suggesting an overall reduction in falls frequency but not recurrence [70].

## 7. Conclusions

Falls in stroke survivors specifically remains an understudied area with no evidence of effective therapy for primary or secondary prevention of falls after stroke. Although the incidence of stroke appears to be reducing in developed nations despite population ageing, the absolute numbers of new strokes per year will continue to rise worldwide. With the burden of stroke and falls set to increase rapidly, more research in this area is now desperately needed. Nevertheless, published evidence so far suggests that risk factors for stroke are similar to that of falls in the general older population. Therefore, investigatory and management approaches for falls among stroke survivors should be similar to existing strategies adopted for general populations. Concerns with regards to blood pressure lowering therapy and anticoagulation among fallers with stroke appear unfounded.
